# Deciphering regulatory variation of THI genes in alcoholic fermentation indicate an impact of Thi3p on *PDC1* expression

**DOI:** 10.1186/1471-2164-15-1085

**Published:** 2014-12-10

**Authors:** Christian Brion, Chloé Ambroset, Pierre Delobel, Isabelle Sanchez, Bruno Blondin

**Affiliations:** INRA, UMR1083, Science pour l’Œnologie, 2 Place Viala, F-34060 Montpellier, France; Montpellier SupAgro, UMR1083, Science pour l’Œnologie, 2 Place Viala, F-34060 Montpellier, France; Université Montpellier 1, UMR1083, Science pour l’Œnologie, 2 Place Viala, F-34060 Montpellier, France

**Keywords:** Thiamine, Pyruvate decarboxylase, Wine yeast, Fermentation, eQTL, Expression regulation

## Abstract

**Background:**

Thiamine availability is involved in glycolytic flux and fermentation efficiency. A deficiency of this vitamin may be responsible for sluggish fermentations in wine making. Therefore, both thiamine uptake and *de novo* synthesis could have key roles in fermentation processes. Thiamine biosynthesis is regulated in response to thiamine availability and is coordinated by the thiamine sensor Thi3p, which activates Pdc2p and Thi2p. We used a genetic approach to identify quantitative trait loci (QTLs) in wine yeast and we discovered that a set of thiamine genes displayed expression-QTL on a common locus, which contains the thiamine regulator *THI3*.

**Results:**

We deciphered here the source of these regulatory variations of the THI and PDC genes. We showed that alteration of *THI3* results in reduced expression of the genes involved in thiamine biosynthesis (*THI11/12/13* and *THI74*) and increased expression of the pyruvate decarboxylase gene *PDC1*. Functional analysis of the allelic effect of *THI3* confirmed the control of the THI and *PDC1* genes. We observed, however, only a small effect of the *THI3* on fermentation kinetics. We demonstrated that the expression levels of several THI genes are correlated with fermentation rate, suggesting that decarboxylation activity could drive gene expression through a modulation of thiamine content. Our data also reveals a new role of Thi3p in the regulation of the main pyruvate decarboxylase gene, *PDC1*.

**Conclusions:**

This highlights a switch from *PDC1* to *PDC5* gene expression during thiamine deficiency, which may improve the thiamine affinity or conservation during the enzymatic reaction. In addition, we observed that the lab allele of *THI3* and of the thiamin transporter *THI7* have diverged from the original alleles, consistent with an adaptation of lab strains to rich media containing an excess of thiamine.

**Electronic supplementary material:**

The online version of this article (doi:10.1186/1471-2164-15-1085) contains supplementary material, which is available to authorized users.

## Background

Controlling the alcoholic fermentation rate of *Saccharomyces cerevisiae* is a key issue for various industrial processes such as wine making, brewing and industrial alcohol production. The glycolytic-fermentation pathway is an essential metabolic process that is linked to the availability of enzyme cofactors, such as NADH/NADPH [1] or vitamins, especially thiamine pyrophosphate (TPP). This vitamin is involved in pyruvate decarboxylation to acetaldehyde.

Glycolytic flux can occur at various intensities depending on strains [2] and environmental conditions. In the wine making process, thiamine levels play a critical role in the outcome of fermentation and a lack of thiamine causes sluggish or stuck fermentation [3, 4]. Grape musts can contain different amounts of thiamine (from 150 to 750 μg/L) depending on grape varieties, agricultural practices and grape processing methods [4]. As a result, the addition of thiamine to musts is a common practice in many cellars. Yeasts actively incorporate this vitamin at the beginning of wine fermentation and no thiamine is left in the medium after six hours [4]. However, *S. cerevisiae* is also able to synthesize thiamine *de novo* from hydroxy-ethylthiazole (HET) and hydroxy-methylpyrimidine (HMP). Nine genes are directly involved in thiamine synthesis: *THI4* (encodes the HET synthase from D-ribulose 5-phosphate (RP), cysteine and glycine), *THI5/11/12/13* (encode HMP synthase from pyridoxal 5-phosphate (PLP) and histidine), *THI20/21* (HMP kinase), *THI6* (HET kinase facilitates the fusion of HMP-HET to thiamine) and *THI80* (thiamine pyrophosphokinase). The expression of these genes requires a high degree of coordination and regulation so that cells can adapt according to thiamine availability. Cellular content of thiamine is sensed by the association of three proteins: Thi2p, Thi3p and Pdc2p, which controls the expression of genes in the thiamine biosynthetic pathway [5]. When the intracellular thiamine level is low, the free form of Thi3p associates with Thi2p and Pdc2p, and the resulting complex activates the transcription by binding to the THI gene promoters [6, 7]. Pdc2p is also known to independently activate the expression of the two pyruvate decarboxylase *PDC1* and *PDC5*[8]. While *PDC1* expression is reportedly not controlled by thiamine level, *PDC5* expression is activated during thiamine deficiency by an unknown mechanism [9].

Activation of THI genes expression has been reported at the end of the growth phase in fermentation, where a decrease in thiamine concentration allows an activation of the pathway. The THI genes were shown to be highly expressed throughout the stationary phase until the end of the process. This is in accordance with a high requirement for thiamine in yeast metabolism [10]. Additionally, enzymatic catalysis has been shown to slowly dismantle the thiamine cofactor as demonstrated with the *Escherichia coli* acetohydroxyacid synthase and *Zymomonas mobilis* pyruvate decarboxylase [11].

There is considerable variation in the ability of yeast to ferment wine as illustrated by the phenotyping of 72 *S. cerevisiae* strains [2]. Some of these differences may arise from modifications in glycolytic enzymes/flux and the availability of cofactors such as thiamine. Variations in the expression of genes involved in thiamine metabolism were observed between the lab strain S288c and the wine strains derivative 59A, which is a sequenced haploid descendent of the strain EC1118. Strain 59A demonstrated stronger expression of THI genes and *PDC5*, but weaker expression of *PDC1* than the lab strain (unpublished data). Uncovering the genetic variations responsible for the different levels of gene expression would enhance our understanding of the evolutionary mechanisms involved.

In yeast, quantitative trait locus (QTL) analyses are now widely used to highlight the allelic effects on phenotypes [12–14]. Variation in regulatory networks have been studied by using both transcriptomic analyses and genetic approaches [15]. We previously carried out a linkage analysis of yeast genes expression under wine alcoholic fermentation conditions [16, 17]. This analysis combined transcriptomic data and phenotype measurements (fermentation rate and metabolite release) of a segregating population from the wine derivative strain (59A) and strain S288c. We searched for expression QTLs (eQTLs) and identified 1465 eQTLs linkages (601 cis-eQTLs and 864 trans-eQTLs) [16].

In this work, a small eQTL hotspot on chromosome 4 was associated with thiamine metabolism without further analysis. Here, we explore variation in this regulatory network. Seven genes involved in thiamine metabolism have a linkage in the hotspot, which maps to the position of *THI3*. Surprisingly, the expression of the major pyruvate decarboxylase encoding gene, *PDC1*, is also impacted by this locus. However, variations in the expression of other THI genes were found to be controlled by other factors. We also suspect that variation in expression of the thiamine transporter, *THI7*, is linked to a mutation in its promoter. For the other THI genes, we observed a strong correlation between expression and fermentation rate.

## Results

### Mapping of expression QTLs over *THI3*location

**Figure 1:**
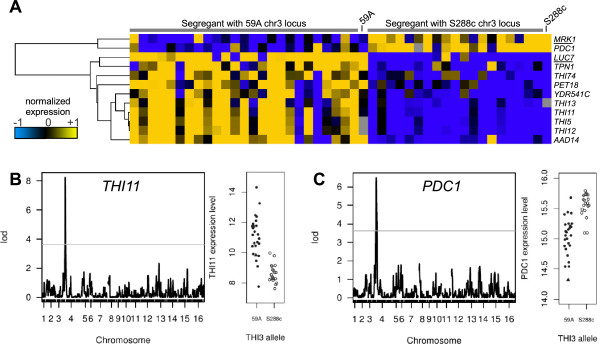
**Hotspot of eQTL mapping on** ***THI3*** **. (A)** Module of 12 genes that display an eQTL on this locus, underlined genes are localized in the locus (cis-eQTL). The genes are sorted by a hierarchical clustering and their expression level has been normalized. **(B)** LODscore pick for *THI11* transcript and *THI3* allele effect on its expression. **(C)** LODscore pick for *PDC1* transcript and *THI3* allele effect on its expression. Grey lines indicate the significant threshold. Expression level corresponds to array log_2_(I).

Our previous study concerning the genetic analysis of expression variations during wine fermentation, in synthetic must [16] revealed a hotspot of 11 eQTLs that mapped to the position 310 kbp on chromosome 4. The genes displaying an eQTL at this locus are listed in Table 1 and the distribution of their expression in the population of segregants is shown in Figure 1A. Several of these genes are involved in thiamine metabolism including thiamine biosynthesis (*THI5/11/12/13*) and thiamine mitochondrial transport (*THI74*), or regulated by thiamine level and have a putative role in thiamine metabolism (*PET18*) [18]. The gene *TPN1,* which also displays an eQTL at this region, encodes a plasma membrane transporter of pyridoxine, a thiamine precursor [19]. In addition, two other genes are controlled by this locus: the aryl-alcohol dehydrogenase, *AAD14*[20], and the major pyruvate decarboxylase, *PDC1*. The four homologous genes *THI5*, *THI11*, *THI12* and *THI13* are involved in the synthesis of the thiamine precursor hydroxy-methylpyrimidine (HMP). *THI5* is absent in the wine derivative strain 59A and was not considered here. The other three genes are identical and could not be dissociated in array transcriptomic analysis given their cross-hybridization, thus they must be considered as a single response.

**Table 1:** **Genes forming a hotspot of eQTL mapping on** ***THI3***

Gene	Name	LODscore	QTL position	sens	Cis-eQTL	Function
YDL087C	LUC7	17,56	chr4:304kbp	59A +	Yes	Essential protein associated with the U1 snRNP complex; splicing factor involved in recognition of 5 splice site; contains two zinc finger motifs; N-terminal zinc finger binds pre-mRNA
YDR438W	THI74	5,32	chr4:306kbp	59A +		Mitochondrial transporter repressible by thiamine
YCR020C	PET18	4,32	chr4:310kbp	59A +		Protein required for respiratory growth and stability of the mitochondrial genome
YJR156C	THI11	7,95	chr4:310kbp	59A +		Protein involved in synthesis of the thiamine precursor hydroxymethylpyrimidine (HMP); member of a subtelomeric gene family including THI5 THI11 THI12 and THI13
YNL332W	THI12	8,55	chr4:310kbp	59A +		Protein involved in synthesis of the thiamine precursor hydroxymethylpyrimidine (HMP); member of a subtelomeric gene family including THI5 THI11 THI12 and THI13
YDL244W	THI13	5,83	chr4:310kbp	59A +		Protein involved in synthesis of the thiamine precursor hydroxymethylpyrimidine (HMP); member of a subtelomeric gene family including THI5 THI11 THI12 and THI13
YDL079C	MRK1	4,23	chr4:310kbp	59A -	Yes	Glycogen synthase kinase 3 (GSK-3) homolog; one of four GSK-3 homologs in *S. cerevisia*e that function to activate Msn2p-dependent transcription of stress responsive genes
YLR044C	PDC1	6,12	chr4:319kbp	59A -		Major of three pyruvate decarboxylase isozymes key enzyme in alcoholic fermentation decarboxylates pyruvate to acetaldehyde
YNL331C	AAD14	5,55	chr4:319kbp	59A +		Putative aryl-alcohol dehydrogenase with similarity to P. chrysosporium aryl-alcohol dehydrogenase; mutational analysis has not yet revealed a physiological role
YDR541C	YDR541C	6,00	chr4:326kbp	59A +		Putative dihydrokaempferol 4-reductase
YGL186C	TPN1	6,26	chr4:326kbp	59A +		Plasma membrane pyridoxine (vitamin B6) transporter; member of the purine-cytosine permease subfamily within the major facilitator superfamily

The QTL region contains *THI3* encoding the thiamine sensor which activates the expression of genes involved in thiamine biosynthesis during thiamine deficiency. The synthetic must provided an excess of thiamine, allowing for cell growth, but it was previously shown that the genes for thiamin synthesis are expressed during the stationary phase of the fermentation [10]. Examination of 59A *THI3* sequence compared to S288c revealed several single nucleotide polymorphisms (SNPs), of which three are non synonymous (Y14C, L344I and D592N). We also observed an insertion of one adenosine in a polyA chain in the C terminal region, causing a frame-shift that altered the protein from amino-acid 605 to the stop codon (Additional file 1). The S288c Thi3p sequence is 609 amino-acids long whereas the 59A version contains 621 amino-acids. We compared the *THI3* sequences of the published genomes from the Sanger Institute [21] or in Genbank. Most *S. cerevisae* strains possess the *THI3* allele found in strain 59A, whereas strain S288c and other lab strains (W303 and BY4742) possess the 609 amino-acids version. *S. paradoxus THI3* sequence display the same number of adenine as 59A and most of *S. cerevisae* strain, suggesting that this version corresponds to the ancestral form of the gene (Additional file 1). The frame-shift at amino-acid 605 in strain S288c may be the cause of the reduced Thi3p function. These mutations are located outside of a proposed thiamine fixation domain (376 to 571 [22]), however they are in a region that might encompass an interacting domain with one of the two transcription factors Thi2p or Pdc2p. The alteration of Thi3p in strain S288c may therefore explain the change in THI gene expression and the generation of the eQTLs hotspot found in our data.

The detection of the *THI11* eQTL and the impact of the *THI3* allele on its expression are displayed on Figure 1B. This illustrates the positive effect of the 59A allele of *THI3* on the expression of the genes involved in thiamine biosynthesis. In contrast, *PDC1* displays an inverse relationship with the *THI3* allele (Figure 1C), its expression being higher in S288c, indicating that the alteration of *THI3* in this strain has a positive impact on *PDC1* transcription. The high LOD score of the *PDC1* eQTL (LODscore = 6.12) suggests that *THI3* allelic variation has an important role in the change of *PDC1* expression. While the *THI3* allele in S288c reduces *THI11* expression, it enhances that of *PDC1*. Our data support the notion that *THI3* negatively impacts *PDC1* expression and the form of Thi3p in S288c is less functional.

### Expression variation of other THI genes independent of *THI3*

**Figure 2:**
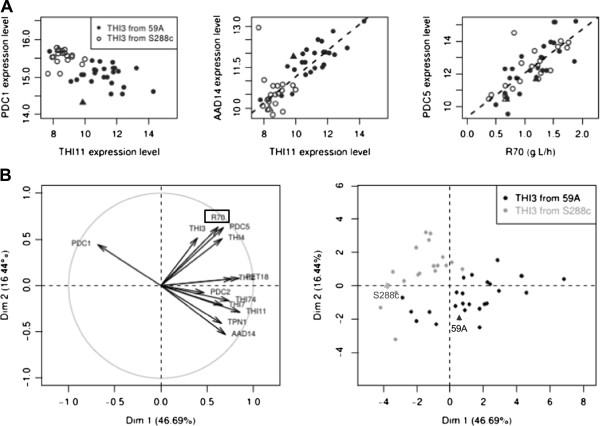
**Overview of correlation between of the genes involved in thiamine biosynthesis and utilization. (A)** Example of correlated expression level: *PDC1* vs *THI11* (negative correlation), *AAD14* vs *THI11* and *PDC1* vs R70. Expression level corresponds to array log_2_(I). Parental strains are indicated with triangles. **(B)** Principal component analysis using as variables the fermentation rate (R70) and expression levels of several genes involved in thiamine biosynthesis and utilization. The first diagonal corresponds to fermentation rate parameters. The second diagonal corresponds to *THI3* origin.

However, as only some of the THI genes are controlled by *THI3* allele, we investigated whether the other thiamine biosynthetic genes displayed linkage to other loci. We did not find any instances of linkage, but instead observed that the expression of most of the genes was correlated with the fermentation rate (at 70% of fermentation progress (R70)). In our previous analysis [16], among the 347 transcripts positively correlated to R70, four were involved in thiamine metabolism (*THI4*, *THI21*, *THI6* and *THI80*). We found that the secondary pyruvate decarboxylase gene, *PDC5*, was also one of the genes whose expression strongly correlated with R70 (Spearman’s correlation coefficient: 0.78, Figure 2A). Interestingly, this enzyme is highly expressed when there is a deficiency of thiamine [9]. Therefore, a tight control of this set of genes by fermentation rate/glycolytic flux may have masked putative linkages to the *THI3* gene.

We performed a principal component analysis of the population of segregants in order to observe the relationship between expression levels of the genes involved. We used the expression of selected THI genes, of the pyruvate decaboxylase genes (*PDC1*, *PDC5* and *PDC2*) and of other genes involved (*AAD14*, *PET18* and *TPN1*), as well as the R70 as variables. The results, displayed in Figure 2B, confirmed that the expression levels are affected by two main factors: fermentation rate and the allelic origin of the *THI3*.

**Figure 3:**
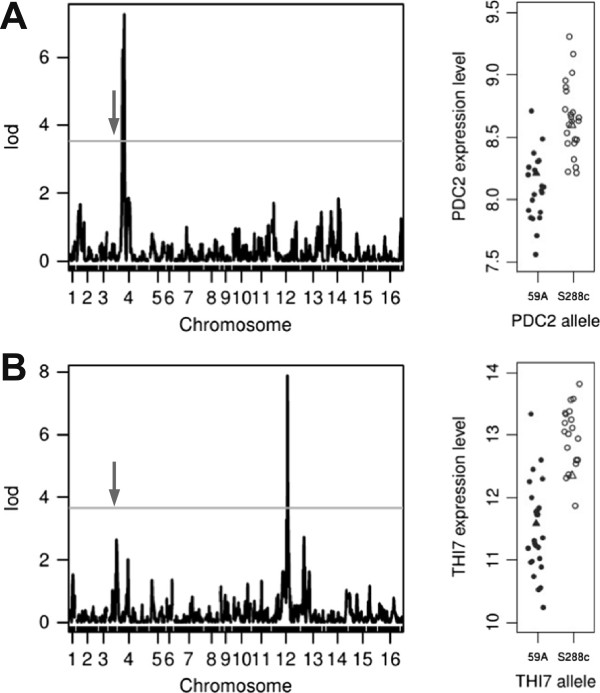
**LODscore pick corresponding to local eQTL; for** ***PDC2*** **(A) and** ***THI7*** **(B) and their allelic effects on expression (right).** Expression level corresponds to array log_2_(I). The arrows indicate the position of *THI3*.

Examination of the eQTLs involving other PDC or THI genes revealed two local cis-regulations as illustrated in Figure 3. The transcription factor *PDC2* displays a cis-eQTL (LOD score of 6.9) but no other gene controlled by *PDC2* displays an eQTL mapping over *PDC2*, meaning that variation in *PDC2* expression level has no major impact on its targets. The *PDC2* allele from S288c is more highly expressed than that of the 59A one; it displays three non-synonymous SNPs in its coding sequence but no SNP in its promoter region, which is consistent with self regulation of its expression.

**Figure 4:**
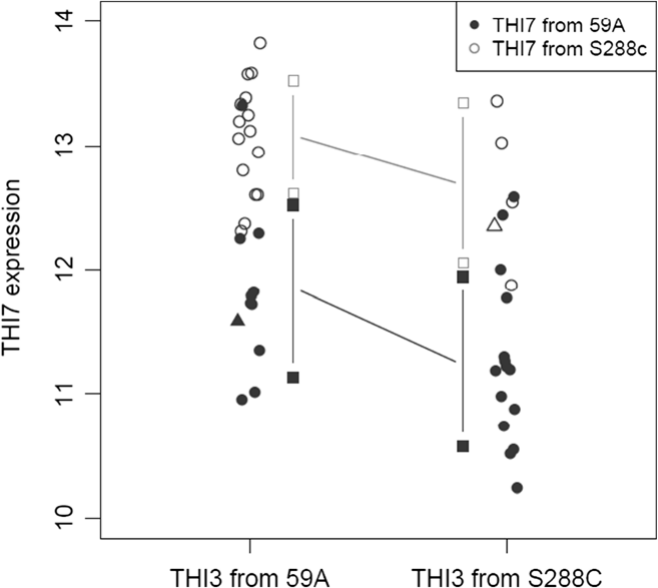
***THI3*** **and** ***THI7*** **double allelic effect on** ***THI7*** **expression.** Expression level corresponds to array log_2_(I). Parental strains are indicated with triangles.

The expression of the thiamine membrane transporter *THI7* (alias *THI10*) also displays a cis-eQTL (LOD score of 7.6). Expression of the lab allele of *THI7* is higher that in the wine yeast. We consistently observed six non-synonymous SNPs in the 59A gene coding sequence and four SNPs in the promoter region. A second LOD peak corresponding to the position of *THI3* (Figure 3B) and the principal component analysis indicate that the *THI3* allele may also have some influence on *THI7* expression. There is indeed an additive effect of the allelic versions of *THI7* and *THI3* origin (Figure 4).

### Functional assessment of the impact of the *THI3*allelic form on PDC and THI gene expression

To confirm the role of *THI3* variation in the control of expression variation of *PDC1* and *THI11/12/13*, an allelic switch of *THI3* was performed in the 59A background, leading to the introduction of the *THI3* gene from S288C (59A *THI3*-S288c, see Materials and Methods). We also carried out the deletion of *THI3* in 59A strain, resulting in a 59A thi3Δ strain, which is auxotrophic for thiamine. To assess the impact on expression, we performed fermentation using the three strains (59A, 59A *THI3*-S288c and 59A thi3Δ) in a synthetic must (SM) medium at 24°C, as previously used for the QTL analysis. The transcription levels at 70% of the fermentation progress were measured by qPCR analysis for three involved genes: *THI11*, *PDC1* and *PDC5* (see Materials and Methods).

**Figure 5:**
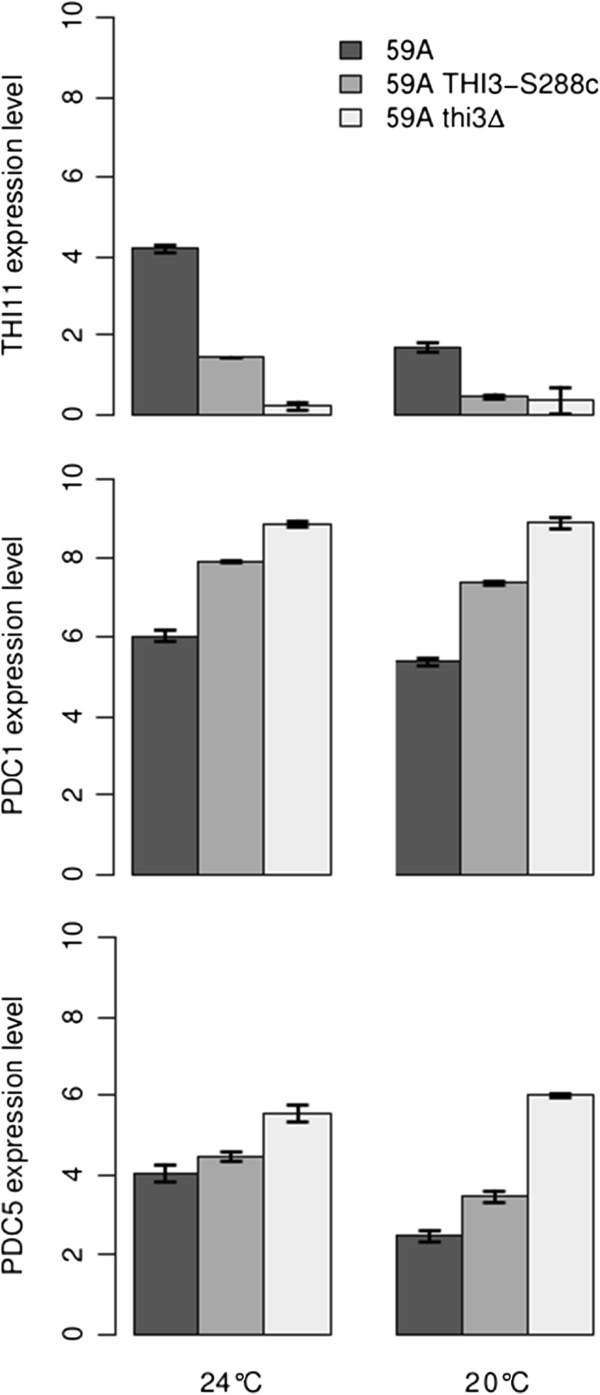
**Expression of the three genes** ***THI11*** **,** ***PDC1*** **and** ***PDC5*** **, measured by rtPCR; depending on** ***THI3*** **background in 59A strain (59A allele, S288c allele and deleted) and on condition (24°C and 20°C).** Expression levels are in log_2_ scale (see Materials and Methods).

The qPCR results are shown in Figure 5. The S288c version of *THI3* (strain 59A THI3-S288c) was associated with an approximately 2.5 log reduction in *THI11* expression. This is consistent with the S288c form of Thi3p having a lower efficiency in THI genes activation. This result confirms that *THI3* is responsible for the linkage of THI genes observed in the eQTL analysis. The S288c allele of *THI3* also triggered an increase in *PDC1* expression of about 2 log (also in agreement with our previous eQTL data), suggesting a direct effect of Thi3p on its expression. In contrast, *PDC5* expression was only slightly impacted by the allelic switch.

The deletion of *THI3* (strain 59A thi3Δ) amplified the effects observed for both *THI11* and *PDC1*: a strong decrease in *THI11* expression and an increase in *PDC1* expression. The lower expression of *THI11* confirms that Thi3p is needed for its expression. Conversely, the higher level of *PDC1* expression triggered by *THI3* deletion supports the idea that Thi3p has a negative impact on its expression. The expression of *PDC5* was also greatly increased by the deletion of *THI3*. In case of thiamine deficiency, *PDC5* is expressed at a higher level but the mechanism, not associated with Thi3p, is still unknown [9, 23]. Without *THI3*, 59A is unable to synthesize thiamine *de novo* and to grow without exogenous thiamine. The increase of *PDC5* expression in strain 59A thi3Δ is probably an indirect consequence associated with a greater thiamine deficiency. In comparison to the deletion effects, the impacts observed for the allelic switch were consistent with our suggestion that S288c allele of *THI3* is less functional.

We carried out a second series of fermentations at 20°C and examined the impact on gene expression. Reducing the temperature allowed us to artificially decrease the rate of fermentation and the glycolytic flux. The expression levels of *PDC5* and *THI11* were lower at 20°C than at 24°C (by 1.5 log and 2 log, respectively) but *PDC1* expression remained constant. These results are probably due to the lower fermentation rate at 20°C and are consistent with the correlation found between R70 and *PDC5* and THI gene expressions levels. This suggests that when there is a weaker glycolytic flux, the demand for thiamine is lower and therefore, we can expect reduced expression of THI genes and of *PDC5*. The same effect of the *THI3* allele on gene expressions was observed in the 20°C fermentation. Therefore our functional analysis was validated in four independent experiments, by testing two genes in two conditions.

### Impact of *THI3*allelic change on fermentation properties

**Figure 6:**
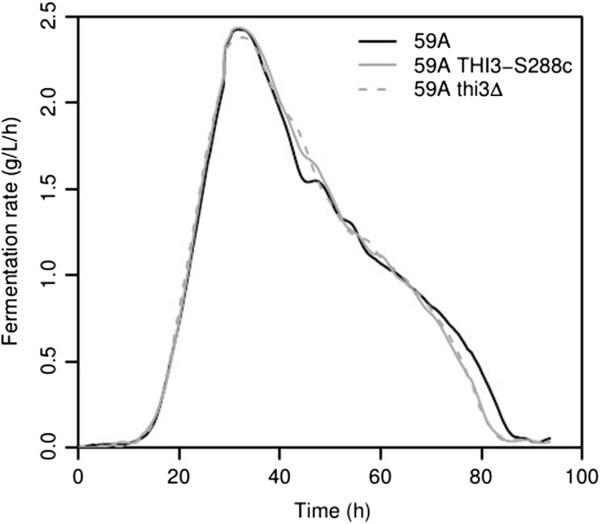
**Comparison of fermentation kinetics of 59A, 59A** ***THI3*** **-S288c and 59A thi3Δ; in the synthetic must standard condition (24°C).** Two additional replicates displayed the same results.

We observed only a very small impact of the *THI3* allelic switch or deletion over fermentation kinetics. In a medium containing thiamine (250 μg/L of thiamine) at 24°C or 20°C, both strain 59A thi3Δ and strain 59A *THI3*-S288c fermented approximately five hours faster than the wild type strain (Figure 6). In a thiamine depleted media, the fermentation remained very slow (CO_2_ production rate 0.25 g/L/h for 0 μg/L of thiamine and 0.5 g/L/h for 25 μg/L of thiamine) and the maximum cell population was lower (50–60.10^9^ cells/L) than when thiamine is available (130.10^9^ cells/L) for the strains 59A and 59A *THI3*-S288c. Although *S. cerevisiae* is able to synthesize thiamine *de novo*, the capacity for endogenous synthesis is insufficient to support growth and fermentation at an equivalent level. The allele origin of *THI3* has no impact on the fermentation rate (data not shown). We also analyzed the growth properties of the two strains (59A and 59A *THI3*-S288c) under aerobic conditions in synthetic must medium without thiamine and found no differences in growth rates or total cell population (data not shown). This indicates that growth capacity and fermentation rate in a thiamine depleted medium is not dependent to the strain ability to activate THI genes.

## Discussion

### Lab yeast evolved on thiamine rich media

**Figure 7:**
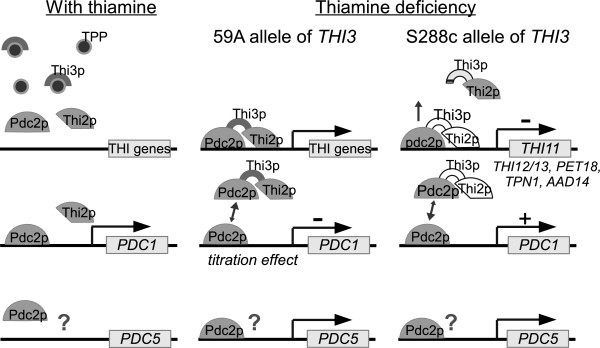
**Putative model of regulation of THI and pyruvate decarboxylase genes; under thiamine deficiency depending on** ***THI3*** **allele.** The regulation mechanism of *PDC5* is still unknown.

A genetic approach allowed us to detect variations in THI gene expression and to link them to mutations in the *THI3* gene. We propose a model of *THI3* allelic effect on the transcriptional regulation of THI genes by thiamine in Figure 7 (based on the model proposed in [6]). In the lab strains, the *THI3* version has a lower efficiency, reducing activation of THI genes. This modification is probably due to the fact that these strains were usually cultivated in rich laboratory media containing an excess of thiamine and are under relaxed selection. Again, the lab strains are not representative of global *S. cerevisiae* behavior, as suggested by Kvitek *et al.*[24]. The laboratory allele of *THI3* is not completely defective, as it does not lead to a thiamine auxotrophy. A similar observation has been made for the reduced function of *ABZ1* in lab strains; this gene is involved in para-amino benzoate biosynthesis, which is unnecessary in rich media [17].

**Figure 8:**
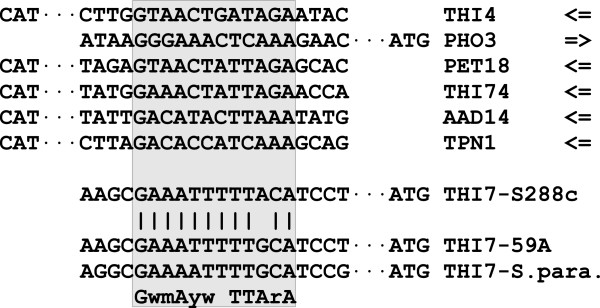
**Thi2p binding site before several THI gene.** The consensus sequence was proposed by Harbinson *et al.*[25].

The eQTL analysis in wine fermentation conditions allowed us to highlight three other genes controlled by *THI3*: *YDR541c* (a putative dihydrokaempferol 4-reductase), *AAD14* (an aryl-alcohol dehydrogenase) and *TNP1* (a pyridoxine transporter). The uptake of pyridoxine may be important for thiamine biosynthesis, as the synthesis of HMP requires pyridoxal 5′-phosphate (PLP). Harbinson *et al.*[25] identified a Thi2p binding domain by genome-wide chromatin immuno-precipitation and we observed this sequence in the promoter region of both *TNP1* and *AAD14* (Figure 8). These observations, and the impact of the *THI3* allele on their expression, suggest a direct role of Thi3p in transcription activation of these genes. Aryl-alcohol dehydrogenases are thought to be involved in alcohol synthesis and act downstream of decarboxylating enzymes. Its control by *THI3* is consistent with the theory that the two sequential reaction steps are coordinated.

Moreover, we observed a negative impact of the ancestral *THI3* allele on *PDC1* expression. Other studies have exclusively used lab strains carrying the weak allele of *THI3*[22, 23]. This may explain why *PDC1* expression has not previously been associated with Thi3p. However, Muller et al. [9], working with strain M5 using two-dimensional gel electrophoresis, reported a diminution of Pdc1p abundance when thiamine was absent from the medium. This response is consistent with the regulatory role of Thi3p described in our study.

The thiamine transporter *THI7* was found to display a local eQTL. This could be due, once again, to an adaptation of *S. cerevisiae* to lab conditions. We studied the promoter sequence of *THI7* and observed an apparition of a Thi2p-binding domain in the sequence of strain S288c due to a SNP not present in the 59A or *S. paradoxus* sequences (Figure 8). This mutation is likely to have a positive effect on *THI7* expression.

However, this hypothesis needs to be validated by functional studies and confirm if these variations are due to positive adaptation or to relaxation. The variability of promoter sequences of the genes involved in thiamine biosynthesis has already been associated with expression variation [26]; which could provide for flexibility across strains to cope with a thiamine deficiency.

### Thi3p control of *PDC1*expression causes a switch from *PDC1*to *PDC5*expression during thiamine deficiency

We suggest that Thi3p does not act on *PDC1* as a true repressor. When there is a deficiency of thiamine, Thi3p binds Thi2p to Pdc2p, triggering THI gene expression. Nosaka *et al.* have studied Pdc2p structure [5] and propose that the interaction with Thi3p triggers a modification in Pdc2p configuration, which activates its transactivation function and the recruitment of the THI promoter. As Pdc2p also triggers the expression of *PDC1*, we suggest that the formation of the Pdc2p-Thi3p-Thi2p complex reduces *PDC1* expression through a titration effect. This hypothetical notion is supported by the fact that *THI3* deletion in strain 59A leads to higher *PDC1* expression than observed in strains 59A or 59A *THI3*-S288c. A lack of complex formation is likely to increase the free form of Pdc2p, which induces *PDC1* expression. In contrast, *PDC5* is expressed more strongly under thiamine deficiency [9] by an unknown mechanism that is independent from Thi3p-Thi2p control [23].

During thiamine deficiency, *PDC5* is expressed strongly but the expression of *PDC1* is likely to be suppressed by the interaction of Pdc2p with Thi2-Thi3p. The reason for this putative enzyme switch remains unclear. Hohmann and Meacock [27] suggest that Pdc5p might have a higher affinity for thiamine than Pdc1p which may help the cell to better cope with thiamine deficiencies. McCourt et al. [11] reported that thiamine was slowly degraded during catalysis reactions by both a bacterial pyruvate decarboxylase and a acetohydroxyacid synthase enzyme. The Pdc1p to Pdc5p shift could allow us to suspect that Pdc5p is better at conserving thiamine whereas Pdc1p optimizes the rate of decarboxylation flux. These hypotheses need to be confirmed by further experiments including the protein-protein interaction measurement for Thi3p as well as the transcription factor and substrate affinity determination for the two pyruvate decarboxylases.

The reduced ability of Thi3p to sense thiamine deficiency in lab strains leads to greater *PDC1* expression; this may have been an adaptive advantage in rich media containing high thiamine concentrations. Under such conditions, *de novo* synthesis of thiamine by THI genes is not critical but the rate of decarboxylation could be optimized. This is relevant to the small improvement of fermentation rate in the strain carrying the lab allele of *THI3* in the thiamine-rich medium.

## Conclusions

Overall, our data indicate that the thiamine gene network regulation was significantly impacted by the adaptation of *Saccharomyces cerevisiae* to thiamine rich laboratory media which masked some regulatory mechanisms and target genes in this species. Therefore, we proposed a putative model of regulation for the main pyruvate decarboxylase in this species, which is dependent on thiamin availability. Our results also suggest that, in conditions of high decarboxylating activity, the impact of catalysis on the thiamine cellular content likely plays a role in the THI genes expression levels.

## Methods

### Yeast strain and culture condition

The two parental strains used in the global analysis are S288c (*MAT*α; *SUC2*, *gal2*) and 59A (*MAT*a; ho), which is a haploid derivative of EC1118 (Lalvin EC-1118, Lallemand, HO/ho), an industrial wine strain [17]. Mutations between the two parental strains are accessible in the GenYeasTrait database previously described (http://genome.jouy.inra.fr/genyeastrait/[17]).

The fermentations were performed for the global analysis, as presented in [16], in a synthetic SM425 medium (with 425 mg/L of assimilate nitrogen pH3.3 and previously describes as SM300 [28]) which mimics natural grape must. Except when indicated, this synthetic medium contained 250 μg/L of thiamine (Sigma T-4625). The same medium and method was used to analyze the constructed strain (59A, 59A *THI3*-S288c and 59A thi3Δ). For the fermentation and growth of 59A and 59A *THI3*-S288c on SM425 without thiamine, the preculture was performed in shake flasks containing 30 mL of SM425 without thiamine at 28°C.

### Strain construction

*THI3* allelic switch in 59A was obtained in two steps: 1) a deletion of *THI3* in 59A using loxP-kanMX-loxP (pUG6 [29]) and 2) an allelic switch by replacement of the kanMX cassette by *THI3* sequence form S288c DNA. Primer sequences for cassette amplification and verification were obtained from Euroscarf. The transformants were selected on YEPD supplemented by G418 (200 μg/L). Primers for the allelic switch are displayed in Table 2. Reinserted *THI3* clones were selected on synthetic minimum medium without thiamine (YwoT, see Additional file 2). The loss of the kanMX cassette was confirmed by PCR and by the absence of growth on YEPD supplemented with G418.

**Table 2:** **Primers used**

Experiment	Aim	Name	sens	5′-3′ sequence
*THI3* allelic switch	kanMX insert	THI3_i_f	Forward	GAAAGAACATAACTACTAAAACGCACCGTCGTCATTCTGAAGATGTTTCGTACGCTGCAGGTCGAC
		THI3_i_r	Reverse	GGTAATCATGAGGGTCCCTGGTAGTAGGGCGGAGAGATCAGATCAGCATAGGCCACTAGTGGATCTG
	THI3-S288c insert	THI3_A	Forward	TGCATTTGTCTTCTGCACACA
		THI3_D	Reverse	TACAAAGTTGCGTGGTATCATCTTA
qPCR	SCR1	sCR1_Cf	Forward	TCTGGTGCGGCAAGGTAGTT
		sCR1_Cr	Reverse	CACCTTTGCTGACGCTGGAT
	UBC6	UBC6_q_F	Forward	GGCTACAAAGCAGGCTCACAA
		UBC6_q_R	Reverse	TGGAGGGTTTTCCACCATCA
	THI11	THI11-qF	Forward	GGTGAATGGTTCGTCCAACAA
		THI11-qR	Reverse	CAAGGCCCGTGGTTTCC
	PDC1	PDC1-qF	Forward	CAGCACCCAAGGTAGCACCA
		PDC1-qR	Reverse	AAACCACTTTCCCAAACAACACC
	PDC5	PDC5-qF	Forward	AAGACTTGGACGATAGCGTATACATC
		PDC5-qR	Reverse	CCAGCTAGAGTTCCAATTACCAAGT

### eQTL linkage analysis

Details on the transcriptomic profiles for the global analysis were provided in a previous publication [16]. This profiles were obtained at 70% of the fermentation progress. The Cy3-labeled cRNAs were hybridized on Agilent gene expression microarrays 8x15k with the one-color method (Agilent Technologies, Santa Clara, CA, USA). Array design is based on ID 016322 completed with the 39 genes from the new regions of EC1118 [30] and available on GEO under the accession number GPL16012. The array pictures were analyzed on a GenePix 4000B laser Scanner (Axon Instruments) with the GenePix PRO7 software. The complete array data set is available on the Gene Expression Omnibus database (http://www.ncbi.nlm.nih.gov/geo, global analysis: GSE41025).

Data normalization and statistical analyses were performed using R 2.13.1 software and the limma package ([31–34]). Normalization was done using the quantile method considering the whole array data set (55 arrays). The normalized LOG_2_ of the spot-median intensity was used as the quantitative evaluation of gene expression (5.3 corresponding to background signal, 16 to spot saturation).

The individual genotyping method is described in a previous publication [17] on Affymetrix YGS98 microarrays. The marker map was designed according to a selection inspired by Brem *et al.* 2002 [15]. This resulted in a map of 2140 markers that we completed with 46 markers localized in areas of low markers density [16]. These markers were based on single nucleotide polymorphism detection using Illumina veracode technology.

The linkage analysis was performed using the R/qtl package [35]. The normal model with a Haley-Knott regression method was used resulting in logarithm of odds (LOD) score for each marker and pseudo-marker every 2.5 cM (7.5kbp, interval mapping method). An interval estimate of the location of each eQTL was obtained as the 1-LOD support interval: the region in which the LOD score is within 1 unit of the peak LOD score. To calculate the false discovery rate of 0.1 at the LOD score threshold of 4, the permutation was performed 20 times, and the average number of transcripts showing linkage was used.

### Quantitative real-time PCR

The fermentations were performed in synthetic must (SM) medium at a temperature of 24°C, as previously used for the QTL analysis. At 66gCO_2_/L release (70% of fermentation progress), 10^9^ cells were sampled, pelleted, washed with DEPC-treated water and frozen in methanol at -80°C. Total RNA extractions were performed (as described in [36]) using the TRIzol® reagent (Gibco BRL, Life Technologies), then purified by isopropanol precipitation and the RNeasy kit (Qiagen). Quality and quantity of RNA were controlled at each step by spectrometry (NanoDrop 1000, Thermo Scientific).

The cDNA was produced by reverse transcription with SuperScript III Reverse Transcriptase (Invitrogen 18080-093, 60 min at 50°C). A 1 in 10 dilution of the resulting cDNA was used for the real-time qPCR assays with gene-specific primers (Table 2) and Power SYBR Green PCR Master Mix (P/N: 4367659 Applied Biosystems) in an ABI7300 qPCR machine. The qPCR was performed in three technical replicates. Expression levels were calculated in LOG_2_ using a calibration range of gDNA for each primer pair then measured relative to *UBC6* expression for each condition. A global correction of the data was performed, taking the lower replicate expression level as 0, for *THI11* expression in 59A thi3Δ, as this strain is not able to express this gene. The good stability of the results was controlled by the *SCR1* expression level which did not change between conditions (data not shown).

### Availability of supporting data

Transcriptomic row data are available in Gene Expression Omnibus database (global analysis: GSE41025, *PDR8* allelic switch analysis: GSE41738). Other supporting data are include in the Additional files 1 and 2.

## Electronic supplementary material

Additional file 1: **Conservation of the adenosine insertion in the THI3 sequence of the** ***S. cerevisiae*** **strains.** (PDF 73 KB)

Additional file 2: **YNB without thiamine (YwoT) medium composition.** (PDF 42 KB)

## Abbreviations

QTL: Quantitative Trait Locus
eQTL: expression QTL
cis-eQTL: “local” eQTL located in less than 40kbp of the controlled gene
trans-eQTL: “distant” eQTL located at more than 40kbp of the controlled gene
R70: CO_2_ release rate at 70% of the fermentation progress
SNP: Single Nucleotide Polymorphism
PDC: Pyruvate decarboxylase
TPP: Thiamine pyrophosphate (or diphosphate)
HET: Hydroxy-ethylthiazole
HMP: Hydroxy-methylpyrimidine
RP: D-ribulose 5-phosphate
XP: D-xylulose 5-phosphate
PLP: Pyridoxal 5-phosphate
qPCR: quantitative polymerase chain reaction
LOD: Logarithm of odds.
