# Supplementary material for: Deciphering regulatory variation of THI genes in alcoholic fermentation indicate an impact of Thi3p on PDC1 expression
Source: BMC Genomics. 2014 Dec 10;15(1):1085. doi: 10.1186/1471-2164-15-1085 (PMC4299793; doi:10.1186/1471-2164-15-1085)
Supplement: Supplementary file 1 — Additional file 1: Conservation of the adenosine insertion in the THI3 sequence of the S. cerevisiae strains. (PDF 73 KB) [file 12864_2014_6896_MOESM1_ESM.pdf]

```
_cttctaaaaaaaaatcaagttggatactga  S288c
_L  L  K  K  N  Q  V  G  Y  *
_cttctaaaa-aaaatcaagttggatactgatctgatctctccgccctactaccagggaccctcatga  EC1118
_L  L  K  -K  I  K  L  D  T  D  L  I  S  P  P  Y  Y  Q  G  P  S  *
_cttctaaaa-aaaatcaagttggatactgatctgatctctccgccctactaccagggaccctcatga  59A
_cttctaaaa-aaaatcaagttggatactgatctgatctctccgccctactaccagggaccctcatga  RM11-1a
_cttctaaaaaaaaatcaagttggatactga  W303
_cttctaaaaaaaaatcaagttggatactga  SGRP_DBVPG6040
_cttctaaaa-aaaatcaagttggatactgatctgatctctccgccctactaccagggaccctcatga  SGRP_YS4
_cttctaaaa-aaaatcaagttggatactgatctgatctctccgccctactaccagggaccctcatga  SGRP_YS2
_cttctaaaa-aaaatcaagttggatactgatctgatctctccgccctactaccagggaccctcatga  SGRP_YJM978
_cttctaaaaaaaaatcaagttggatactga  SGRP_YIIc17_E5
_cttctaaaa-aaaatcaagttggatactgatctgatctctccgccctactaccagggaccctcatga  SGRP_L_1374
_cttctaaaa-aaaatcaagttggatactgatctgatctctccgccctactaccagggaccctcatga  SGRP_DBVPG1788
_cttctaaaa-aaaatcaagttggatactgatctgatctctccgccctactaccagggaccctcatga  SGRP_BC187
_cttctaaaa-aaaatcaagttggatactgatctgatctctccgccctactaccagggaccctcatga  SGRP_378604X
_cttctaaaa-aaaatcaagttggatactgatctgatctctccgccctactaccagggaccctcatga  SGRP_322134S
_cttctaaaa-aaaatcaagttggatactgatctgatctctccgccctactaccagggaccctcatga  SGRP_YS9
_cttctaaaa-aaaatcaagttggatactgatctgatctctccgccctactaccagggaccctcatga  SGRP_YJM981
_cttctaaag-aaaatcaagttggatactgatctgatctctccgccctactaccagggaccctcatga  SGRP_DBVPG1853
_cttctaaaa-aaaatcaagttggatactgatctgatctctccgccctactaccagggaccctcatga  SGRP_273614N
_cttctaaaa-aaaatcaagttggatactgatctgatctctccgccctactaccagggaccctcatga  SGRP_UWOPS05_227_2
_cttctaaaa-aaaatcaagttggatactgatctgatctctccgccctactaccagggaccctcatga  SGRP_UWOPS05_217_3
_cttctaaaa-aaaatcaagttggatactgatctgatctctccgccctactaccagggaccctcatga  SGRP_NCYC361
_cttctaaaa-aaaatcaagttggatactgatctgatctctccgccctactaccagggaccctcatga  SGRP_YPS60
_cttctaaaa-aaaatcaagttggatactgatctgatctctccgccctactaccagggaccctcatga  SGRP_Y9
_cttctaaaa-aaaatcaagttggatactgatctgatctctccgccctactaccagggaccctcatga  SGRP_K11
_cttctaaaa-aaaatcaagttggatactgatctgatctctccgccctactaccagggaccctcatga  SGRP_NCYC110
_cttctaaaaaaaaatcaagttggatactga  WU_YJM653
_cttctaaaaaaaaatcaagttggatactga  WU_FL100
_cttctaaaa-aaaatcaagttggatactgatctgatctctccgccctactaccagggaccctcatga  WU_YJM280
_cttctaaaa-aaaatcaagttggatactgatctgatctctccgccctactaccagggaccctcatga  WU_M22
_cttctaaaa-aaaatcaagttggatactgatctgatctctccgccctactaccagggaccctcatga  WU_CLIB215
_cttctaaaa-aaaatcaagttggatactgatctgatctctccgccctactaccagggaccctcatga  WU_YJM421
_cttctaaaa-aaaatcaagttggatactgatctgatctctccgccctactaccagggaccctcatga  WU_T73
_cttctaaaa-aaaatcaagttggatactgatctgatctctccgccctactaccagggaccctcatga  WU_CBS7960
_cttctaaaa-aaaatcaagttggatactgatctgatctctccgccctactaccagggaccctcatga  WU_I14
_cttctaaaa-aaaatcaagttggatactgatctgatctctccgccctactaccagggaccctcatga  WU_YPS163
_cttctaaaa-aaaatcaagttggatactgatctgatctctccgccctactaccagggaccctcatga  WU_YPS1009
_cttctaaaa-aaaatcaagttggatactgatctgatctctccgccctactaccagggaccctcatga  WU_YJM269
_cttctaaaa-aaaatcaagttggatactgatctgatctctccgccctactaccagggaccctcatga  WU_T7
_cttctaaaa-aaaatcaaattagacactgacatgatctctccgccctattatcagggtcctccatga  S. pardoxus
```

Additional file 1. Conservation of the adenosine insertion in THI3 sequence of the S. cerevisiae strains and impact on protein sequence. SGRP: strain sequences from Saccharomyces Genome Resequencing Project. WU: strain sequences from Washington University.
