# Supplementary material for: Deciphering regulatory variation of THI genes in alcoholic fermentation indicate an impact of Thi3p on PDC1 expression
Source: BMC Genomics. 2014 Dec 10;15(1):1085. doi: 10.1186/1471-2164-15-1085 (PMC4299793; doi:10.1186/1471-2164-15-1085)
Supplement: Supplementary file 2 — Additional file 2: YNB without thiamine (YwoT) medium composition. (PDF 42 KB) [file 12864_2014_6896_MOESM2_ESM.pdf]

Table S1: YNB without thiamine (YwoT)

Component	Formula		final concentration
Dextrose	$C_6H_{12}O_6$		20g/L
Potassium Phosphate	KH_2PO_4		1g/L
Magnesium Sulfate	$MgSO_4$, $7H_2O$		0.5g/L
Sodium Chloride	$NaCl$		0.1g/L
Calcium Chloride	$CaCl_2$, $2H_2O$		0.1g/L
Ammonium Chloride	NH_4Cl		5g/L
**Mineral nutrients (stock solution)**		**stock concentration**	**1mL/L**
Manganese Sulfate	$MnSO_4$, H_2O	4g/L	
Zinc Sulfate	$ZnSO_4$, $7H_2O$	4g/L	
Copper Sulfate	$CuSO_4$, $5H_2O$	1g/L	
Potassium Iodure	KI	1g/L	
Cobalt Chlorure	$CoCl_2$, $6H_2O$	0.4g/L	
Boric Acide	H_3BO_3	1g/L	
Ammonium heptamolybdate	$(NH_4)_6Mo_7O_{24}$	1g/L	
**Vitamines (stock solution)**		**stock concentration**	**10mL/L**
Myo-Inositol	-	2g/L	
Calcium Pantothenate	-	0.15g/L	
Nicotinic Acide	-	0.2g/L	
Pyridoxine	-	0.025g/L	
Biotine	-	0.3mg/L	
